# Sphingolipid Metabolism Correlates with Cerebrospinal Fluid Beta Amyloid Levels in Alzheimer’s Disease

**DOI:** 10.1371/journal.pone.0125597

**Published:** 2015-05-04

**Authors:** Alfred N. Fonteh, Cora Ormseth, Jiarong Chiang, Matthew Cipolla, Xianghong Arakaki, Michael G. Harrington

**Affiliations:** Molecular Neurology Program, Huntington Medical Research Institutes, 99 N El Molino Ave, Pasadena, California, United Sates of America; Louisiana State University Health Sciences Center, UNITED STATES

## Abstract

Sphingolipids are important in many brain functions but their role in Alzheimer’s disease (AD) is not completely defined. A major limit is availability of fresh brain tissue with defined AD pathology. The discovery that cerebrospinal fluid (CSF) contains abundant nanoparticles that include synaptic vesicles and large dense core vesicles offer an accessible sample to study these organelles, while the supernatant fluid allows study of brain interstitial metabolism. Our objective was to characterize sphingolipids in nanoparticles representative of membrane vesicle metabolism, and in supernatant fluid representative of interstitial metabolism from study participants with varying levels of cognitive dysfunction. We recently described the recruitment, diagnosis, and CSF collection from cognitively normal or impaired study participants. Using liquid chromatography tandem mass spectrometry, we report that cognitively normal participants had measureable levels of sphingomyelin, ceramide, and dihydroceramide species, but that their distribution differed between nanoparticles and supernatant fluid, and further differed in those with cognitive impairment. In CSF from AD compared with cognitively normal participants: a) total sphingomyelin levels were lower in nanoparticles and supernatant fluid; b) levels of ceramide species were lower in nanoparticles and higher in supernatant fluid; c) three sphingomyelin species were reduced in the nanoparticle fraction. Moreover, three sphingomyelin species in the nanoparticle fraction were lower in mild cognitive impairment compared with cognitively normal participants. The activity of acid, but not neutral sphingomyelinase was significantly reduced in the CSF from AD participants. The reduction in acid sphingomylinase in CSF from AD participants was independent of depression and psychotropic medications. Acid sphingomyelinase activity positively correlated with amyloid β_42_ concentration in CSF from cognitively normal but not impaired participants. In dementia, altered sphingolipid metabolism, decreased acid sphingomyelinase activity and its lost association with CSF amyloid β_42_ concentration, underscores the potential of sphingolipids as disease biomarkers, and acid sphingomyelinase as a target for AD diagnosis and/or treatment.

## Introduction

Late onset Alzheimer’s disease (AD) is a neurodegenerative brain disorder that affects over 5 million Americans and is projected to increase in prevalence with an aging population [1]. Without an understanding of the causes and means of preventing AD, the economic and social ramifications will seriously strain healthcare systems [2]. AD is characterized in the early phase by beta-amyloid deposition and subsequently by neurofibrillary tangles [3]. These protein abnormalities are associated with synaptic dysfunction and brain atrophy. Neuroinflammatory processes can initiate abnormal processing of amyloid proteins and alter AD metabolic pathways [4]. As lipids constitute a major portion of the dry mass of the brain, changes in their composition can alter neuronal function [5,6]. Moreover, certain signaling lipids are inflammatory [7,8], others resolve brain inflammation [4,8], and some induce apoptosis of neurons [9,10].

Sphingolipids (SP) are a lipid class that includes sphingomyelin (SM), ceramides (Cer), dihydroceramides (dhCer), and their glycosylated, sulfated or phosphorylated derivatives [11]. SPs are the focus of this study because their roles are central to many physiological processes that are altered in AD. SM is found in myelin sheaths of neurons and lipid rafts of cell membranes. Degradation of SM may cause disturbances in action potentials and synaptic dysfunction associated with neurological disorders [12,13]. The major enzymes that regulate SM levels are sphingomyelin synthase and sphingomyelinases (SMase). SMases degrade SM to form Cer and choline. Acid (aSMase), neutral (nSMase), and alkaline forms of the enzyme exist [14,15]. nSMase is membrane-bound while aSMase is secreted or localizes to lysosomal compartments [16]. An increase in SMase expression or activity may cause excessive breakdown of SM. On the other hand, low levels of SMase such as in Niemann-Pick Disease may result in the buildup of SM in major organs including the brain, resulting in irreversible neurological damage [16,17].

Catabolism of SM forms Cer, an important lipid second messenger associated with apoptosis [17–19]. Cer also promotes beta-amyloid biogenesis and studies link it to oxidative stress [20,21]. Since Cers regulate neurotransmitter release and synaptic vesicle fusion [22,23], their altered levels may lead to synaptic dysfunction.

Secretase enzymes involved in amyloid precursor protein processing are localized in SP-rich lipid rafts and amyloid transport is associated with SM levels [24,25]. Moreover, the autophagocytic clearance of amyloid peptides involves both nSMase and aSMase activities [26], and perturbation of SP metabolism is related to learning and memory [27,28].

It is clear from these roles that SP metabolism will be altered in AD, although many details have not been characterized. SPs have been characterized in several CSF studies and have been shown to change in prodromal AD [29–32]. However, it is not known whether SPs in CSF are derived from neuronal vesicles or other brain components. The discovery of abundant nanoparticles in CSF that include synaptic vesicles and large dense core vesicles [33] allowed us to test this hypothesis by determining the SP composition of brain-derived nanoparticles (NP) compared to the interstitial fluid-derived supernatant fluid (SF) fraction. We show that SPs are differentially distributed in cell membranes and in supernatant fluids, and change with aSMase activity and beta amyloid in study participants that are cognitively normal (CN) compared with mild cognitive impairment (MCI) or AD.

## Materials and Methods

### Materials

HPLC solvents were purchased from VWR (West Chester, PA). Tris base and Tris acid, ammonium acetate and butylated hydroxyl toluene (BHT) were purchased from Sigma (St Louis, MO). Internal standard (IS) [1,2-diundecanoyl-*sn*-glycero-3-phosphocholine, PC(11:0/11:0], 1-palmitoyl-2-oleoyl-*sn*-glycero-3-phosphocholine (PC(16:0/18:1), and SP standards (bovine SM, Cer, dhCer, GlcCer) were purchased from Avanti Polar Lipids (Alabaster, AL).

### Clinical methods and cerebrospinal fluid collection

All protocols were approved by the Institutional Review Board of the Huntington Hospital (Pasadena, CA) and conformed to the Helsinki Declaration. Informed consents were signed by each study participant or by their legal representative. CN participants (n = 70) with mean age (SD) of 77 (7) years, ranging in age from 63–89 years (61.5% females), were asymptomatic with a Clinical Dementia Rating [34] score of zero, and their classification methods were reported recently [35]. Briefly, the participants were characterized after a full medical intake, including medication history, the Geriatric Depression Scale (GDS, 15 items), and neurological examination; they were classified as CN if they had neuropsychological measures within one standard deviation of the mean for their age and education according to published normative values, and did not meet the criteria for MCI [36] or dementia [37]. MCI (n = 40) with mean age (SD) of 77 (7), age range 60–91 years (60% females), and clinically probable AD (n = 29) with mean age (SD) of 77 (10), age range 47–91 years (55.2% female) were diagnosed in study participants by current clinical criteria [37,38]. CSF samples were collected between 8.00–10.00 am after an overnight fast and within a month of neuropsychological assessment. Total protein, cell counts, and glucose were determined immediately, and the remainder of the CSF was stored at -80° C in 1 ml aliquots.

### Determination of protein content

CSF fractions were diluted using PBS and the concentrations of protein determined using a fluorescence-based Quant-iT Protein Assay detection Kit (Invitrogen, Eugene, OR) using bovine serum albumin (BSA, 0–500 ng) for standardization.

### Amyloid β_42_ and total tau in CSF

Aβ_42_ and total tau levels in CSF were measured using a sandwich ELISA kit (Innotest β-amyloid_(1–42)_ and Innotest hTAU-Ag, Innogenetics, Gent, Belgium) following the manufacturer’s recommended protocol. All assays were performed in the same week from CSF aliquots that had never been re-frozen, were collected within a 2 year period and stored in a monitored freezer that never warmed above -75°C. CSF samples were analyzed in duplicate, with 8 standards in duplicate, by an investigator blinded to diagnosis.

### APOE genotype

Blood peripheral lymphocytes and standard methods for APOE genotyping were used [39].

### Cerebrospinal fluid fractionation and sphingolipid extraction

CSF was fractionated into SF and NP fractions using ultracentrifugation. NP pellets were washed once and re-suspended in phosphate buffered saline [33]. To reduce contaminants, borosilicate glass tubes were used for extraction of all lipid samples. Glass test tubes were washed with 2 mL CHCl_3_/CH_3_OH (1:1), heated at 100°C for 1 hr., rinsed with 2 mL CH_3_OH, followed by heating at 100°C for 15 min before use. IS [5 ng PC(11:0/11:0)] was added to 1 ml SF or NP suspended in 1 ml water containing 0.9% formic acid and 1 M NaCl before lipid extraction using a modified Bligh and Dyer method [40]. Methanol used in the extraction contained 0.2 mg/ml BHT. All extraction procedures were performed at room temperature. The lipid-rich chloroform layer containing SPs (SM, Cer, and dhCer) was dried under a stream of N_2_ and used for liquid chromatography-mass spectrometry (LC-MS) studies.

### Liquid chromatography of sphingolipids

LC was performed using an HP-1100 system equipped with an autosampler, a column oven maintained at 35°C, and a binary pump system [41]. SPs were separated using a TSK-Gel Amide-80 Column (2.0 x 150 mm) with a solvent system of acetonitrile/isopropanol/20 mM ammonium formate (20:73.6:6.4, v/v/v, Solvent A), and isopropanol/20 mM ammonium formate (20:80 v/v, Solvent B) at a flow rate of 0.25 ml/min. The solvent composition was maintained at 100% A for 5 minutes to elute Cer and dhCer. Subsequently, the percentage of solvent B was increased to 20% over 20 min to elute SM.

### Electrospray ionization tandem mass spectrometry of sphingolipids

SPs from the LC column were positively ionized using electrospray ionization and detected using a triple quadrupole mass spectrometer (TSQ Quantum from Thermo Fisher Scientific). The MS parameters were optimized for best detection of SP species eluting from the LC at different solvent mixtures at 0.25 mL/min. The MS was operated with a spray voltage of 4.5 kV, heated capillary temperature of 300°C, with nitrogen (50 units) and argon (5 units) as the sheath gas and the auxiliary gas, respectively. Cer species (RT 1.6 min) was obtained using neutral loss scan of 264 with acquisition mass range of 460–1200. dhCer species (RT 1.8) was acquired using neutral ion loss of 266 with a m/z range of 470–1200. PC(11:0/11:0) IS (RT ~14.9 min) was acquired using selected reaction monitoring (SRM) of m/z 595 (precursor ion) to 184 (product ion) [41]. SM (RT 20.5) was acquired using precursor scanning m/z 184 with m/z range of 630–920. Baseline resolution of PC from SM gives us confidence that we are identifying the right lipid species in our samples, S1 Fig SP scans were optimized with respect to collision energies and acquired data integrated using Xcalibur software (Thermo Fisher Scientific). SP amounts were determined using standard curves obtained from running authentic standards (0–50 ng) containing PC(11:0/11:0) IS.

### Mass spectrometry data analyses

SP peaks were integrated using the Qual Browser module of the Xcalibur software (Thermo Fisher, San Jose, CA) and normalized to PC(11:0/11:0) used as an internal standard (IS). For calibration curves, 0–50 ng SP standards (Cer (C18:0), GlcCer (C18:0), dhCer (C18:0) and bovine SM) and a fixed amount of IS [5 ng PC(11:0/11:0)] were run separately and standard curves of SP amounts versus SP/PC(11:0/11:0) obtained. From the ensuing standard curve, SP levels in SF (ng/ml CSF) or NP (ng/ml CSF equivalent) were calculated.

Using the spectra function of the Qual Browser software, 15 representative SM molecular species were quantified. SP species were chosen from the Lipid Maps Databases (http://www.lipidmaps.org/tools/ms/sphingolipids_batch.html). Extracted ion intensities of identified peaks were compared to the internal standard [PC(11:0/11:0)] and amounts estimated using standard curves for SM. Our approach for isolating isobaric SM species [41], calculation of SP levels, and rationale for not using LIPID MAPS sphingolipid internal standards [42] are described in S1–S3 Methods, S2 and S3 Figs.

### Sphingomyelinase activity assays

nSMase activity was measured with the Amplex Red SMase Assay Kit (Molecular Probes). CSF samples were diluted to a concentration of 10 μg protein/100 μL. Samples were preincubated at 37°C for 5 minutes, and then 100 μL assay cocktail solution containing 2U/mL horseradish peroxidase, 0.2 U/mL choline oxidase, 8 U/mL alkaline phosphatase and 0.5 mM SM was added to each sample for a total volume of 200 μL. Fluorescence was measured at excitation 545 nm and emission at 590 nm for 1 h at 37°C.

For aSMase assay, CSF protein (10 μg) was incubated with SM in acetate buffer (pH 5.0) for 1 h at 37°C. The pH of the solution was increased to 8.0 using 100 mM Tris/HCl buffer before the addition of the assay cocktail solution to detect released choline phosphate as described above for nSMase. For a combined two-step assay, aSMase assay was determined under acid conditions and the change in fluorescence representing nSMase activity monitored for 1 hour after increasing the pH to 8.0 as described above.

### Statistical analyses

To avoid any bias in our study, CSF samples were randomly processed under identical extraction conditions, and the technician was blinded to diagnosis throughout data acquisition and analyses. SP levels and amount of SM molecular species in SF and NP are presented as the mean ± SEM for all clinical groups. Wilcoxon test of paired *t-test* data was used to compare the proportion of SM species in SF and NP fractions. Mann—Whitney *U* tests were used to compare SP and SMase levels between different cognitive groups. For multiple comparisons, we used ANOVA (Kruskel Wallis) with Dunn's multiple comparisons tests. Receiver operating characteristic (ROC curves) were obtained by comparing CN controls to either MCI or AD samples. Confounding variables were evaluated using non-parametric ANCOVA. All analyses were performed using Graph Pad Prism software (La Jolla, CA) or SAS (Cary, NC), and significance was set at *P* value < *0*.*05*.

## Results

### Demographic data and CSF biomarkers

We recruited 70 CN study participants, 40 MCI subjects, and 29 subjects with probable AD. The AD group differed from the MCI and CN groups by education and APOE genotype, well—recognized for AD cohorts [43]. GDS scores were higher in the AD participants compared with the CN group; the use of anti-depressants was not different between the clinical groups; the use of total psychotropic medications (mainly from anti-cholinesterase therapies) was greater in the AD compared to both MCI and CN groups (Table 1) The other demographic and CSF parameters are well matched between the groups. CSF Aβ_42_ levels were higher (*p* < 0.05) in the CN (722 ± 299) and MCI (754 ± 268) groups than in AD (506 ± 230). CSF total tau levels were lower (*p* < 0.05) in the CN (273 ± 149) and MCI (265 ± 168) groups than in AD (473 ± 222).

**Table 1:** GDS scores and usage of anti-depressants and psychotropic drugs by clinical group.

Clinical group	GDS (0–15 scale) Mean (SD)	Anti-depressant use	Psychotropic use
CN, n = 70	1.2 (1.9)	16%	19%
MCI, n = 37	1.5 (1.9)	24%	16%
AD, n = 33	2.4 (1.8)*	36%	67%**

*GDS score of the AD group is higher than the CN group, ANOVA *p* < 0.05.

**The use of psychotropic drugs is greater in the AD cohort than both CN and MCI groups, ANOVA *p* < 0.0001.

### Sphingolipid content of CSF fractions

**Fig 1:**
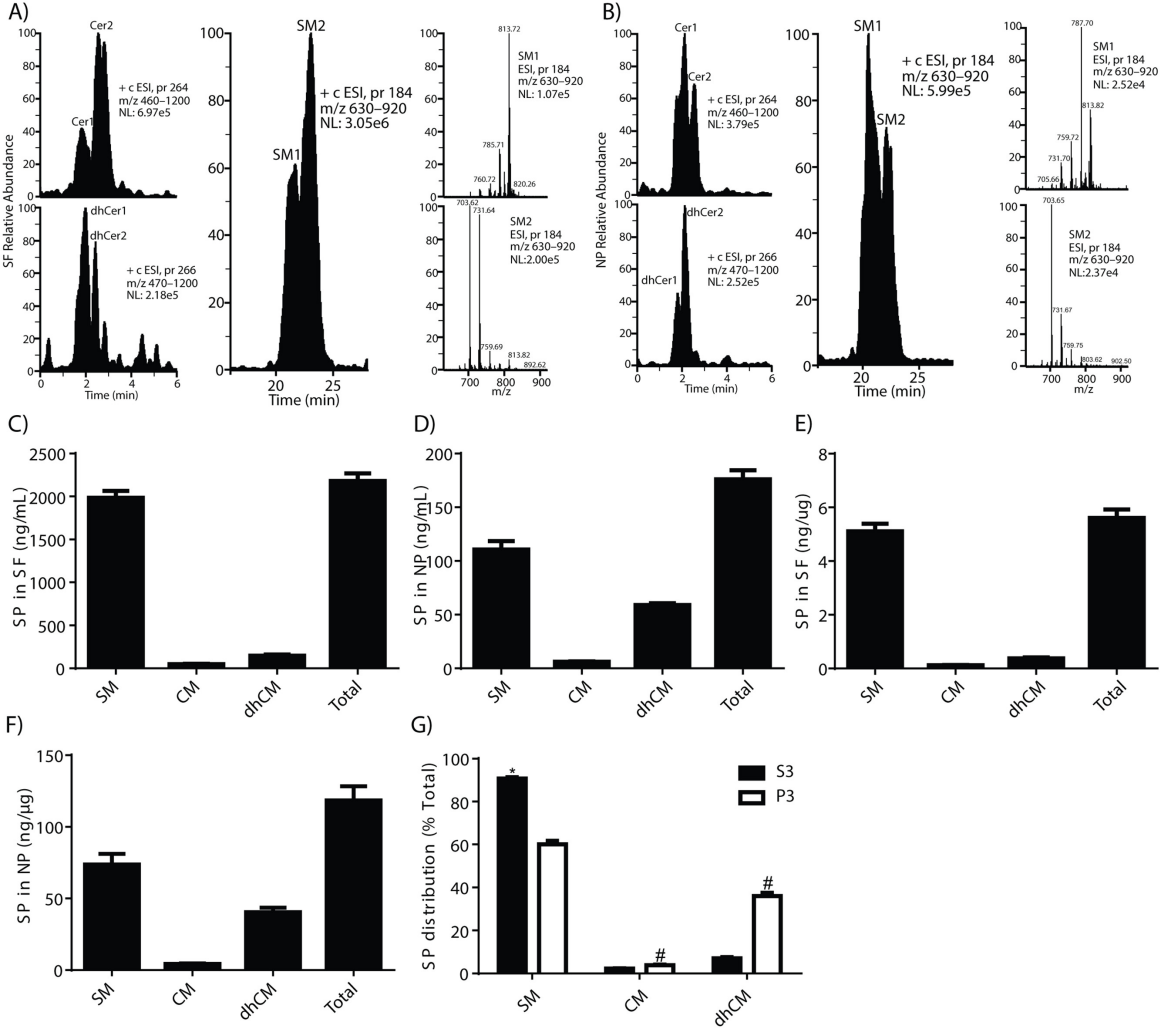
Identification and quantification of sphingolipids. SPs extracted from CSF fractions were detected using LC-MS/MS. Fig 1 shows the scan specific chromatograph for Cer, dhCer, SM, and the spectra of early- (SM1) and late- (SM2) eluting SM peaks for SF (A) and NP (B), respectively. Fig 1C and 1D are SP levels in SF and NP normalized to the volume of CSF while Fig 1E and 1F are levels of SP in SF and NP normalized to the amount of protein in each fraction. Fig 1G shows the percent distribution of SPs in SF and NP. The asterisk (*) denotes SP distribution in SF > NP (*P* < 0.05) and # denotes SP distribution in NP > SF (*P* < 0.05). These data are the mean ± SEM for n = 70 (SF) and n = 67 (NP), respectively.

To determine if there were differences in the origin or metabolism of SP between the SF and NP fractions, we first examined the SP composition of these fractions from the CN group. Initial identification of SP classes and species in CSF fractions from the CN study participants allowed subsequent targeted analyses and comparison with MCI and AD samples. PC(11:0/11:0) served as a useful internal standard since it was resolved from SP classes and gave a linear relationship with each class (Table 2), S2 Fig Scan and retention time-specific analyses revealed three major SP classes in SF from the CN group: SM, Cer and dhCer (Fig 1A). On the LC column, all three SP classes were partially resolved into two peaks; SM1 and SM2 for SM; Cer1 and Cer2 for Cer; and dhCer1 and dhCer2 for dhCer. The high peak intensity of the SM peak allowed us to examine its spectral composition. SM1 appears to be predominantly made of higher m/z molecular species compared to SM2 (Fig 1A). The same LC-MS/MS analysis of NP from CN participants shows a similar profile of SPs (Fig 1B). However, the ratio of SM1 to SM2 in the NP fraction (1.41 ± 0.32) was higher (*P < 0*.*005*) than that in SF (0.49 ± 0.19) suggesting that the NP fraction had a higher proportion of higher m/z species than the SF fraction. Cer and dhCer also partially resolved into two peaks in the CSF fractions but their intensity was not high enough for quantitative analyses for most CSF fractions. Examination of the most intense samples showed that Cer peaks (Cer1 and Cer2) had a similar distribution to the SM peaks (Cer2 > Cer1 in SF and Cer1 > Cer2 in NP), while the distribution for dhCer was reversed, with a higher dhCer2/dhCer1 in the NP than in the SF fraction (Fig 1A and 1B).

**Table 2:** MS scan parameters, recovery, limit of detection, and linearity of SP standards.

SPs	MS Scan precursors,(m/z)	LOD (ng)	R^2^, mean ± SEM, (95% CI), n	% Recovery (95% CI), n
Cer (C18:0)	264	0.47	0.97 ± 0.03(0.89–1.04), 5	65.5 ± 2.8(58.6–72.3), 6
GlcCer (C18:0)	264	0.70	0.97 ± 0.02(0.89–1.02), 3	88.7 ± 4.1(71.2–106.2), 3
dhCer (C18:0)	266	2.5	0.96 ± 0.02(0.89–1.02), 5	71.0 ± 2.0(65.8–76.2), 6
SM (Bovine brain)	184	0.16	0.95 ± 0.02(0.93–0.97), 4	88.7 ± 6.9(70.9–106.4), 6
PC(C11:0/C11:0)	SRM 184	N/A	N/A	N/A

Abbreviations: LOD, limit of detection; CI, confidence interval

Using standard curves, we quantified SP classes in SF and NP (Fig 1C and 1D). The SF and NP fractions had similar sphingolipids but with different proportions (Fig 1C and 1D, respectively). When normalized to the protein content of each fraction (Fig 1E and 1F), total SP levels were over 20-fold higher in the NP fraction (118.4 ± 9.9, n = 69) than the SF (5.6 ± 0.3, n = 69). The different proportions of SPs in CSF fractions were evident when comparing the SM to Cer ratio in SF (46.8 ± 2.7, n = 69) with SM/Cer in NP (18.4 ± 1.3, n = 69) or the percentage of total for each SP class (Fig 1F). While SM levels were higher in SF than in NP (*P < 0*.*05*), proportions of Cer and dhCer were significantly enriched in the NP compared to the SF fraction (Fig 1G) (*P < 0*.*05*).

#### Sphingomyelin molecular species in CSF fractions

**Fig 2:**
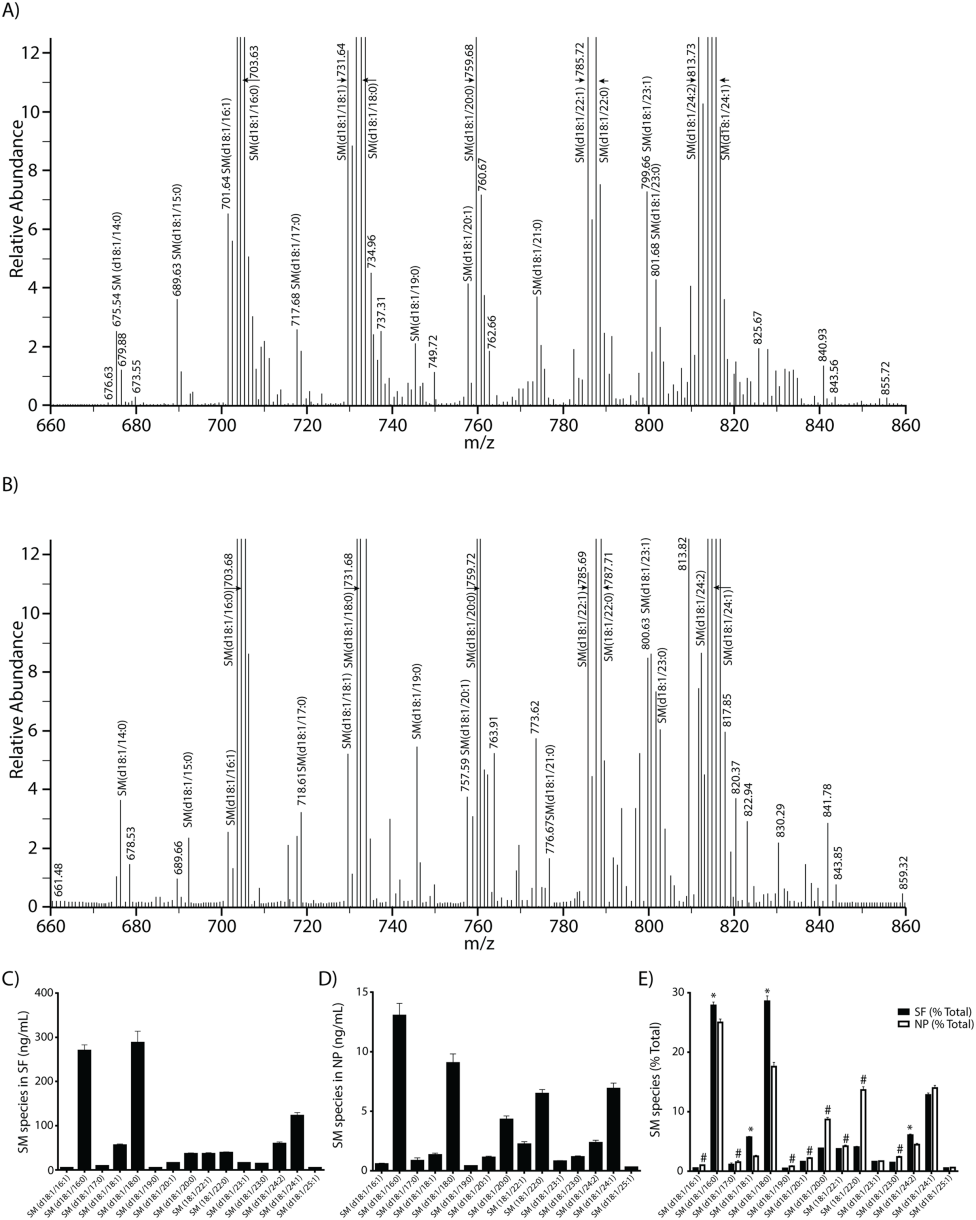
Spectra and levels of sphingomyelin species. SM species from LC-MS/MS were processed using the spectral feature of the Qual Browser software. Fig 2 shows representative spectra of SM species identified in SF (2A) and NP (2B). Amounts of 15 SM molecular species were determined in SF (2C) and NP (2D) and expressed as a percent of total in each CSF fraction (2E). The *P* values were obtained using a paired t-test. The asterisk (*) on Fig 2E denotes SM species whose proportions are higher in SF than NP (*P < 0*.*05*) and # denotes SM molecular species whose proportions are higher in NP than SF (*P < 0*.*05*). The unmarked species have similar proportions in both fractions (*P > 0*.*05*). These data are the mean ± SEM for SF (n = 70), and NP (n = 67).

Since SM is highly expressed in SF and NP, we were able to extract and quantify the most intense molecular species in CSF fractions. Spectral analyses of SM showed several distinct species in SF (Fig 2A) and NP (Fig 2B). Detailed analyses revealed at least 45 SM species that were spectrally resolved in our LC-MS/MS runs, S1 Table. Using extracted ion chromatography, we were able to reliably quantify a representative set of SM species in SF and NP fractions, S4 Fig As expected, ion intensities of each species were higher in SF than NP when normalized to CSF volume, S4 Fig Input of extracted masses into the Lipid Maps MS prediction tool for sphingolipids resulted in the identification of SP species in SF and NP fractions, S2 and S3 Tables. We next calculated levels of 15 different SM species in CSF fractions that were not burdened by isobaric interference as described in the Methods. Fig 2C and 2D show the levels of these species in SF and NP, respectively. Next we compared levels of SM species in CSF fractions by expressing the quantities of each species as a percentage of the total in each fraction (Fig 2E). Wilcoxon tests of paired *t* test data revealed that some SM species [SM(d18:1/16:0), SM(d18:1/18:1), SM(d18:1/18:0) and SM(d18:1/24:2)] were higher in SF than NP; others [SM(d18:1/16:1), SM(d18:1/17:0), SM(d18:1/19:0), SM(d18:1/20:1), SM(d18:1/20:0), SM(d18:1/22:1), SM(d18:1/22:0), SM(d18:1/23:0) were higher in NP than SF, while others [SM(d18:1/23:1), SM(d18:1/24:1), SM(d18:1/25:1)] had similar proportions in each fraction. The lower m/z species were more likely to be higher in SF than NP while the higher m/z species were higher in NP than SF. To confirm our extracted ion chromatography (EIC) data that SF and NP were enriched with SM species, we used multiple reaction monitoring (MRM) to identify the major SM species in these fractions. Targeted MRM studies showed a similar distribution of SM species as the EIC data (data not shown). Together, these data showed the abundance and variety of SM species in CSF from older cognitively normal adults, and their different levels between the SF and NP fractions.

#### Ceramide species in CSF fractions

SPs have modified variants including hydroxylated, glucosylated, and sulfated species (http://lipidlibrary.aocs.org/Lipids/sphingo.html). The Cer peaks Cer1 and Cer2 are composed of several molecular species, S5 Fig We identified glycosphingolipid (GlcSP) species in the SF fraction, S5 Fig and S4 Table, and in the NP fraction, S5 Fig and S5 Table, that varied considerably between different CSF samples,

#### Dihydroceramide species in CSF fractions

Similar to Cer, several dhCer variants are known [44]. dhCer molecular species were different in SF and NP fractions, S5 Fig, S6 and S7 Tables.

### Changes in sphingolipids based on neurocognitive classification

**Fig 3:**
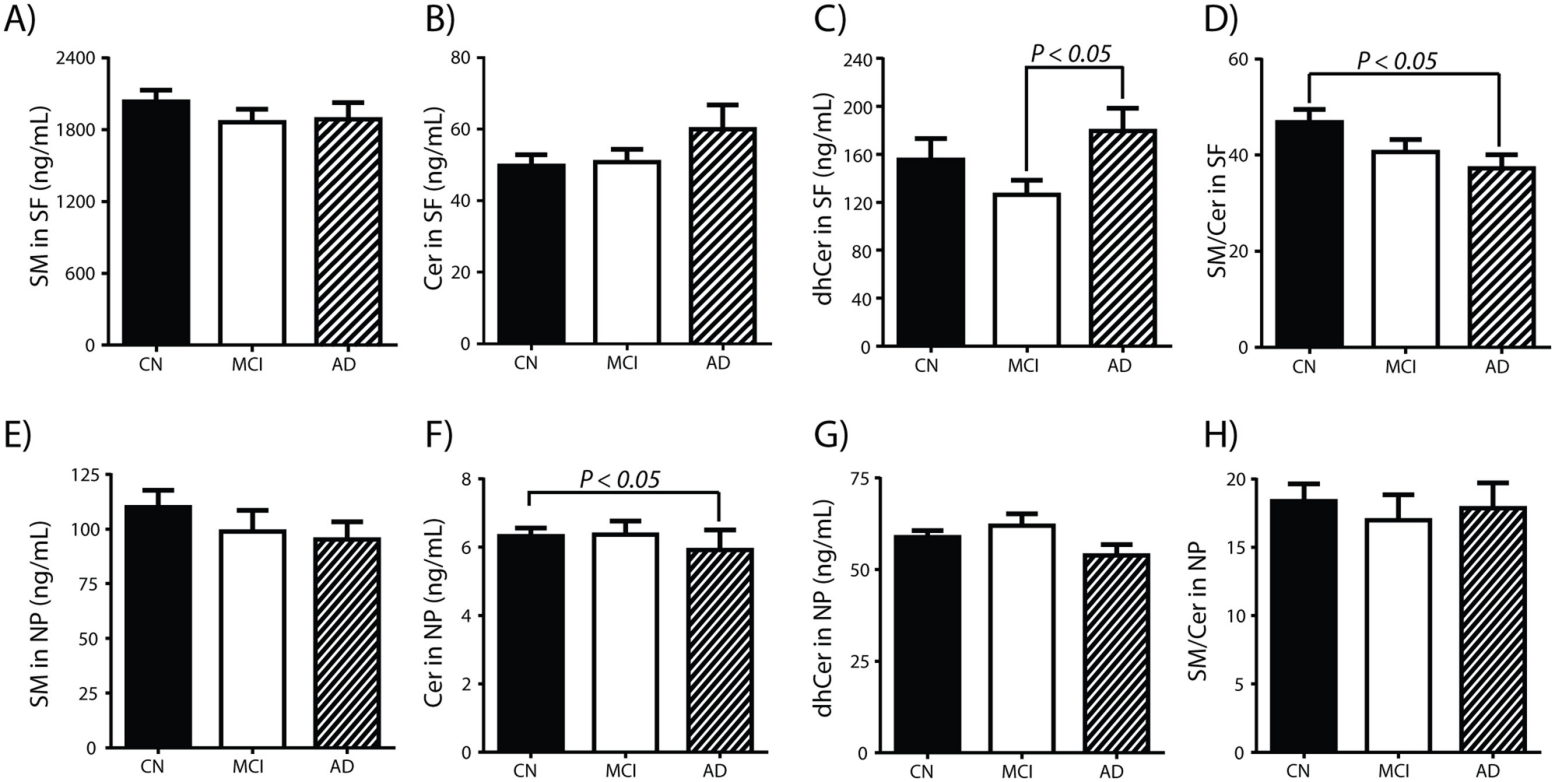
Sphingolipid levels are altered in Alzheimer’s disease. Levels of SPs in SF or NP were determined using LC-MS/MS. Fig 3A, 3B, 3C, and 3D are the levels (mean ± SEM) of SM, Cer, dhCer, and the ratio SM/Cer in SF for CN, MCI or AD subjects. Fig. 3E, 3F, 3G, and 3H are the levels (mean ± SEM) of SM, Cer, dhCer, and the ratio SM/Cer in NP for CN, MCI or AD subjects. Group comparisons were performed using Kruskal Wallis test and non-parametric comparisons using Mann Whitney test and differences between cognitive groups indicated when *P < 0*.*05*.

We next quantified the abundance of SP classes in SF from CN, MCI and AD. Although mean SM levels in all three clinical groups were similar, we noticed a slight decrease in MCI and AD (Fig 3A). Levels of Cer and dhCer were not different when comparing CN to MCI (Fig 3B and 3C). In contrast, there was an increase in Cer and dhCer in AD (Fig 3B and 3C). The slight decrease in SM (-7.3% for AD) concomitant with the increase in Cer (21.8% for AD) in CSF was more evident when we compared the ratio SM/Cer in CN to that of AD (Fig 3D). A similar study examining SP changes in NP fractions revealed a decrease in the mean levels of SM (Fig 3E), Cer (Fig 3F, *P < 0*.*05*) and dhCer (Fig 3G) in AD compared with CN such that the ratio SM/Cer did not change (Fig 3H).

**Fig 4:**
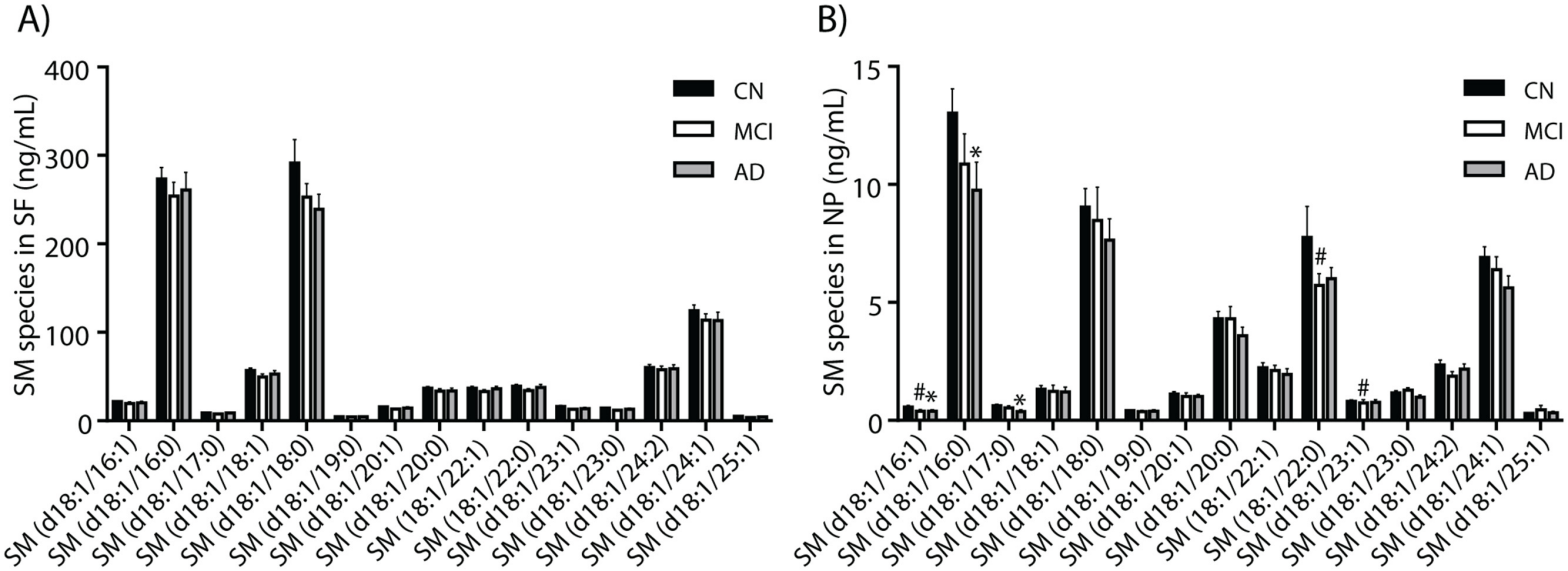
Some sphingomyelin species decrease in Alzheimer’s disease. After LC-MS/MS of SF and NP, SM species were extracted and their intensities normalized to PC(11:0/11:0) as an internal standard. Amounts of each molecular species were determined in SF (4A), in NP (4B) in CSF fractions from CN (n = 69), MCI (n = 38) and AD (n = 28). The *P* values were obtained using Kruskal Wallis test with Dunn’s multiple comparison and Mann-Whitney *U* test. Data are the mean ± SEM and * denotes values of *P < 0*.*05* for CN compared with AD and # denote *P < 0*.*05* for CN compared with MCI.

We determined the levels of SM molecular species in the three clinical groups in the SF (Fig 4A) and NP (Fig 4B) fractions. In the NP fraction, three molecular species [SM(d18:1/16:1), SM(d18:1/16:0), SM(d18:1/17:0)] were significantly depleted in AD compared with CN (Fig 4B). Three SM species [SM(d18:1/16:1), SM(d18:1/22:0), SM(d18:1/23:1)] were lower in the NP fraction of MCI compared with CN while no differences in the levels of SM species were detected when comparing MCI with AD (Fig 4B). Together, these data showed an increase in Cer in the supernatant fluid fraction and a decrease in Cer and three SM species in the particulate fraction in AD compared with CN study participants.

### Sphingomyelinase activity in cerebrospinal fluid

**Fig 5:**
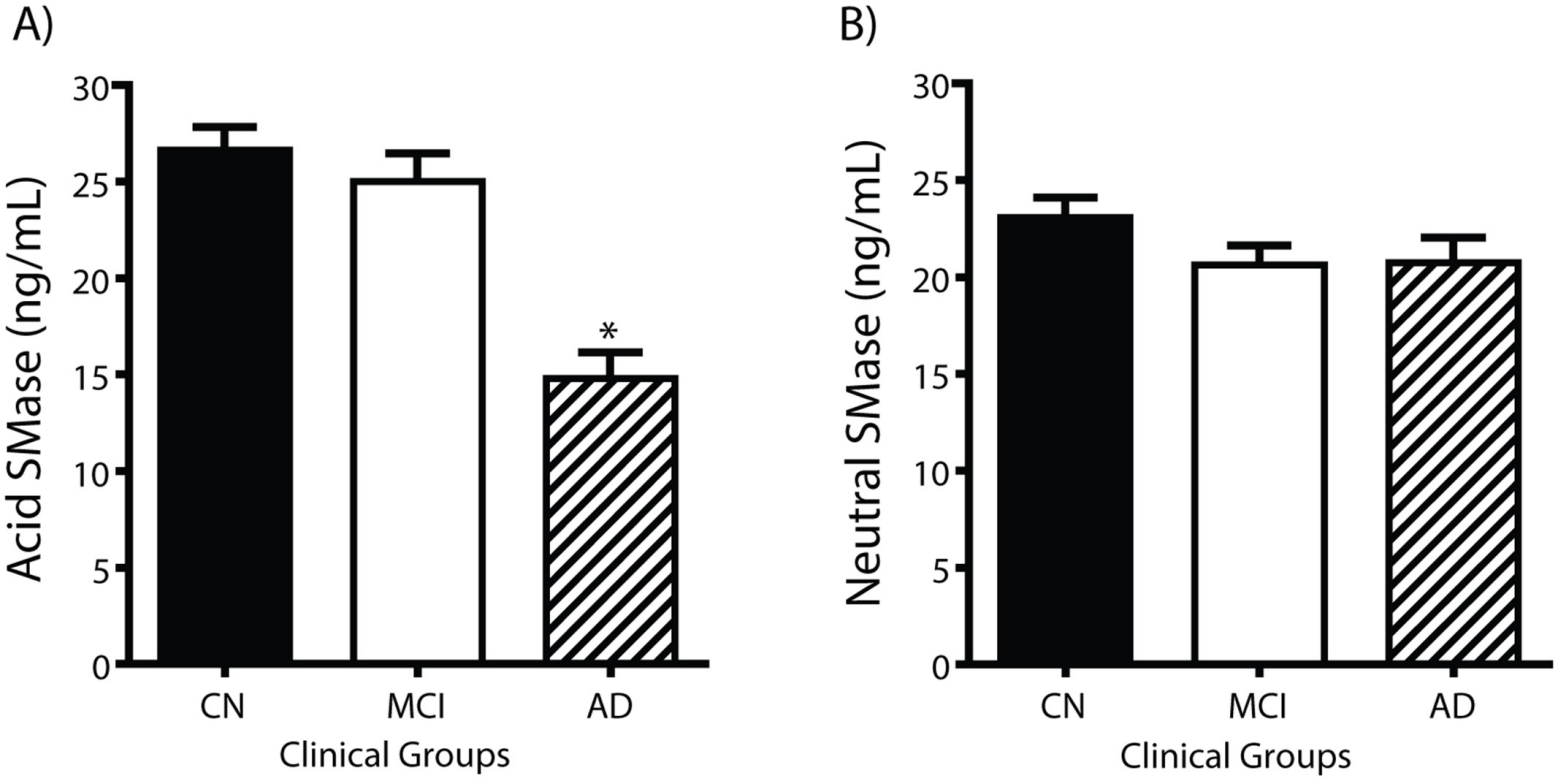
Sphingomyelinase activity decreases in Alzheimer’s disease. aSMase (5A) and nSMase (5B) activities were measured in CSF using fluorescent assays. The data are expressed as the mean ± SEM of the relative fluorescent unit (RFU) per minute using 10 μg protein per assay for CN (n = 70), MCI (n = 40), and AD (n = 29). The asterisk (*) denotes values of *P < 0*.*05* compared with CN.

Since SMase activity can account for the decrease of SM in CSF fractions concomitant with the increase of Cer in SF, we measured the activity of the major SMase isoforms, aSMase and nSMase, in CSF. Both aSMase and nSMase activities were detected in CSF from CN study participants (Fig 5). Compared with CN (26.6 ± 1.2 RUF/min, n = 69, range = 45.48), mean aSMase activity decreased in CSF from MCI (-6.1%, range = 37.46) and in AD (-44.4%, range = 26.75, *P < 0*.*05*) (Fig 5A). Mean nSMase activity also decreased in MCI (-10.5%) and AD (-10.1%) compared with CN (Fig 5B). Spearman correlation studies indicate that aSMase and nSMase activities do not correlate with SP levels in all cognitive groups (data not shown). Together, these data suggest that SMase activity in CSF is not related to the depletion of SM and the increase of Cer in the SF fractions in AD, but is consistent with a role in the decreased Cer in the NP fraction.

**Fig 6:**
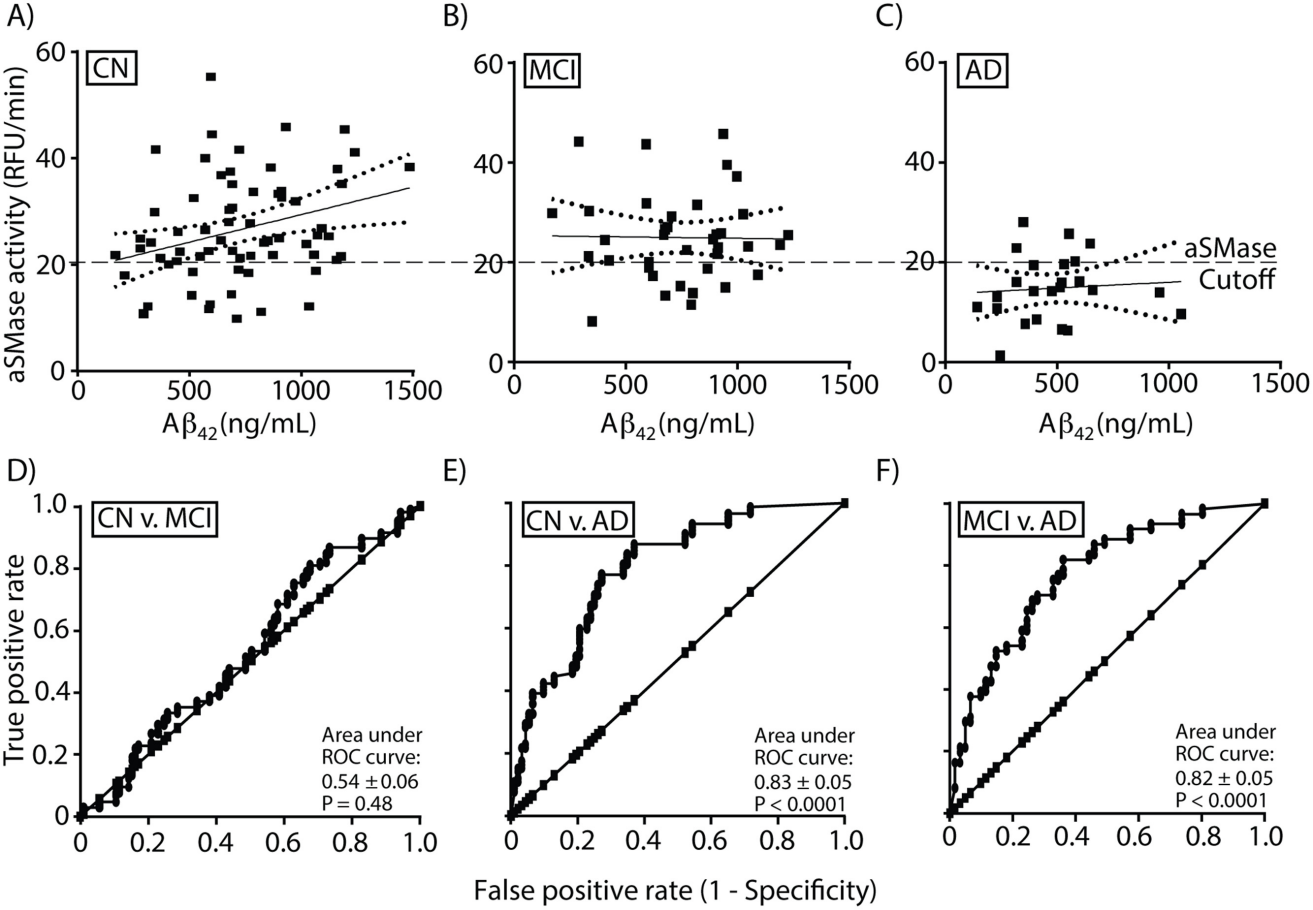
Aβ_42_ correlation with sphingomyelinase activity and ROC curves. Plots of Aβ_42_ levels in CSF versus aSMase activity indicating the linear regression line with 95% confidence interval (dotted line) for CN (6A), MCI (6B), and AD (6C). Spearman r analyses indicate a significant positive correlation between Aβ_42_ and aSMase in CN (*P < 0*.*01*) but not for MCI (*P >0*.*99*) or AD (*P > 0*.*39*). Linear regression analyses show that the slope is significantly non-zero for CN (*P < 0*.*01*) but not for MCI (*P > 0*.*16*) and AD (*P > 0*.*72*). An aSMase activity cutoff value of 20 RFU/min is shown for CN, MCI and AD.

We compared SMase activities with levels of Aβ_42_ or total tau since these enzymes are known to induce Aβ_42_ release or are activated in cell culture studies [45]. aSMase activity positively correlated with Aβ_42_ levels in CSF from CN (Spearman r = 0.32, *p <0*.*01*, n = 69) but not for MCI (Spearman r = 0.0007, *p > 0*.*99*, n = 37) or AD (Spearman r = 0.18, *p > 0*.*18*, n = 24) participants (Fig 6A, 6B and 6C, respectively). In contrast, nSMase activity did not correlate with Aβ_42_ in any of the groups (data not shown). Similarly, total tau levels in CSF did not correlate with aSMase or nSMase activity (data not shown). These data suggest that SP metabolism known to be important in autophagocytosis [26], may be linked to the higher levels of Aβ_42_ found in the CSF from cognitively normal subjects while the lower levels of aSMase may partially account for lower Aβ_42_ levels found in CSF from AD subjects.

Since depression and anxiety have been associated with aSMase activity [46,47], we compared GDS scores with aSMase activity in our clinical groups. GDS scores are not correlated with aSMase activity in AD: Spearmans r = 0.09. *p* = 0.7. aSMase levels for AD on antidepressants (N = 11; mean (SD) = 35.2 (26.6); median = 33.3) do not differ from AD study participants not on antidepressants (N = 13; mean(SD) = 38.8 (13.7); median = 41.1): Kruskal Wallis test *P* = 0.24. SMase levels for AD patients on psychotropics (N = 16; mean (SD) = 37.5 (23.6); median = 37.7) do not differ from AD not on psychotropics (N = 8; mean (SD) = 36.6 (12.1); median = 39.0): Kruskal Wallis test *P* = 0.71.

Using non-parametric ANCOVA’s to examine group differences in SMase activity levels: a) Controlling for GDS, F(3,116) = 2.91, *P* = 0.06; aSMase activity in the AD group was significantly lower than both MCI (*P* = 0.02) and CN (*P* = 0.04) groups; the MCI and CN groups do not differ from one another (*P* = 0.6). b) Controlling for antidepressant medications, F(3,126) = 2.38, *P* = 0.10; aSMase activity in the AD group was significantly lower than the MCI group (*P* = 0.03) and borderline lower than the CN group (*P* = 0.07); the MCI and CN groups do not differ from one another (*P* = 0.5). c) Controlling for psychotropic medications, F(3,126) = 2.77, *P* = 0.07; SMase activity in the AD group was significantly lower than the MCI (*P* = 0.03) and CN (*P* = 0.04) groups; the MCI and CN groups do not differ from one another (*P* = 0.7).

### Acid sphingomyelinase as a CSF biomarker of cognitive function

To determine if aSMase activity can discriminate CN from AD, we performed sensitivity and specificity calculations using a cutoff of 20 RFU/min for low activity. This 20 RFU/min cutoff was chosen such that about 80% of the AD samples had lower SMase activity. For CN, 14 CSF samples were below the cutoff and 55 were above. For MCI, aSMase activities in 10 CSF samples were below and 27 were above 20 RFU/min. Comparing AD and CN, sensitivity and specificity of 79.2% and 79.7% were obtained with positive predictive value of 57.6% and negative predictive value of 91.6%. For AD and MCI, sensitivity and specificity of 79.2% and 73% were obtained with positive predictive value of 65.5% and negative predictive value of 84.4%. Finally, for MCI and CN, sensitivity and specificity of 27% and 79.7% were obtained with positive predictive value of 41.7% and negative predictive value of 67.1%. These data were supported by ROC curves showing that aSMase activity did not differentiate CN from MCI (Fig 6D) but significantly differentiated CN from AD (Fig 6E), and MCI from AD (Fig 6F).

## Discussion

To examine SP metabolism in the brain, we used LC-MS/MS to quantify the major SP classes in CSF fractions of supernatant fluid (SF) and nanometer-sized particles (NP). The major discoveries from our study are: 1) SP composition differs between the SF and NP fractions when normalized to CSF volume or the protein content of each fraction. Relative to protein content, SPs were much more abundant in the NP fraction. We measured proportionally more SM in the SF than the NP fraction. Conversely, the proportions of Cer and dhCer were higher in the NP than the SF fraction. The distribution of SP species also differed between the two fractions. 2) Comparing levels of the major sphingolipids between clinical groups, the SM/Cer ratio increased in the SF fraction in AD compared with CN, while the Cer fraction decreased in the NP fraction when we compared AD to MCI. 3) Levels of three out of 15 low molecular SM species decreased in the NP fraction of AD compared with CN participants. 4) aSMase but not nSMase activity decreased in CSF from AD compared with CN and MCI. aSMase decrease in AD was independent of depression or the use of psychotropic medications. 5) aSMase activity correlated with Aβ_42_ levels in CSF only in CN participants. 6) aSMase levels differentiate CN from AD, and MCI from AD. These results suggest an important potential role of SP compartmentalization in metabolism and neurodegeneration, a possible diagnostic role for aSMase, and implications for candidate targets for AD therapy.

### Compartmentalization of sphingolipid in cognitively healthy participants

Sphingolipids are synthesized in the endoplasmic reticulum with serine and palmitoyl CoA as the main precursors. Our measurements of dhCer, Cer and SM levels in CSF fractions give direct knowledge of the complexity and compartmentalization of SP metabolism. Hannun and Obeid proposed a new paradigm in Cer metabolism in which compartmentalization is based on the activities of over 28 different enzymes [48]. Microarray studies reveal variation in several enzymes that metabolize SPs as a function of brain region [49,50]. Our data support such an enzyme and substrate compartmentalization involving multiple pathways by showing differences in SP composition between different CSF fractions. Whereas SM constitutes most of the SP in the SF fraction, the NP fraction has reduced SM and is significantly enriched with dhCer and Cer. One interpretation of these data is that the NP fraction may be the site for the *de novo* synthesis of SP. Of interest also is the fact that there are different SM species in SF and NP fractions, supporting the notion that there are different metabolic pathways for different sphingolipid pools in the brain

While we show differential distribution of Cer species in CSF fractions, the low abundance, low sensitivity, or low resolving power of our MS does not allow us to identify and reliably quantify these species. Since sulfatide species that are components of myelin sheaths and have been shown to be depleted in AD [51], a comprehensive database including unsaturated, saturated, hydroxylated, glucosylated, and sulfated variants is needed to enable the complete identification of these species in CSF.

### Role of sphingolipid metabolism in AD

SP metabolism has been associated with AD in several studies [52–56]. SPs play important roles in AD by interacting and controlling Aβ_42_ secretion [54,57], and forming signaling molecules involved in cell growth or apoptosis [22]. In our study, we report an increase in Cer in SF and a decrease in the NP fraction in the AD group. The increase in Cer was evident when we examined the SM to Cer ratio in SF and found a lower ratio in AD compared with CN. Since we measured Cer increases in the SF fraction and a decrease in the NP fraction, this may indicate different SP metabolism in interstitial fluid versus brain membranes. Ceramides are formed by several pathways in response to different apoptotic signals [22,58]. Cer increase in the SF fraction may indicate an increase in *de novo* synthesis concomitant with a decrease in SM synthesis. In the NP fraction, decreased Cer may be accounted for by a decrease in the SM pathway and may be related to a decrease in membrane-bound SMase activity. These data suggest that there are different sphingolipid pools or metabolic pathways that change in AD compared with CN participants.

### Sphingomyelinase activity

A major pathway associated with cytokine-induced apoptosis is the enzyme-dependent hydrolysis of SM. Several studies suggest that both aSMase and nSMase can induce apoptosis in cell lines [59,60]. We hypothesized that the changes in SM and Cer measured in CSF were due to an increase in SMase activity. Paradoxically, we measured a significant decrease in aSMase activity in AD compared with CN and MCI, whereas nSMase activity was only slightly decreased, suggesting that the SMase pathway is not directly responsible for the increase in Cer and subsequent decrease in SM/Cer found in SF from our AD participants. SP metabolism is linked to major depression and anxiety disorders [46,47] and antidepressant therapy has been shown to decrease aSMase activity [61,62]. Our analysis demonstrated that the decrease of aSMase in AD we report here was not due to any confounding effect from depression, its treatment, or from the use of psychotropic medications, notably the greater use of anticholinesterases by AD participants.

The decrease in SMase activity, however, may relate to the decrease in the Cer content of the NP fraction. Recent studies suggest that there are many enzymatic pathways that control Cer levels [48]. An increase in SM synthase activity has recently been associated with Aβ_42_ secretion of cells and the regulation of protein trafficking [63,64]. Therefore, the SM decrease we detected in both CSF fractions may indicate a decrease in SM synthase activity and this may relate to the decreased secretion of Aβ_42_in AD. Increased Cer may also result from *de novo* synthesis when an enzyme such as DES that is responsive to increased oxidative stress is activated [65]. Therefore, any increase in Cer levels may be due to changes in the expression or activities of several enzymes in AD.

### Sphingomyelinase activity and Aβ_42_ secretion

SPs are linked to exosome formation and have recently been shown to promote Aβ_42_ secretion, endocytosis, and plasma membrane repair. Whereas the increase in SM synthesis stimulates exosome formation, the increase in nSMase activity prevents exocytosis [66]. Secreted exosomes containing Aβ_42_ are thought to be metabolized by glial cells in a phosphatidylserine-dependent pathway that involves lysosomal processing [57]. aSMase is a lysosomal enzyme that may play a role in autophagocytosis [67,68].

**Fig 7:**
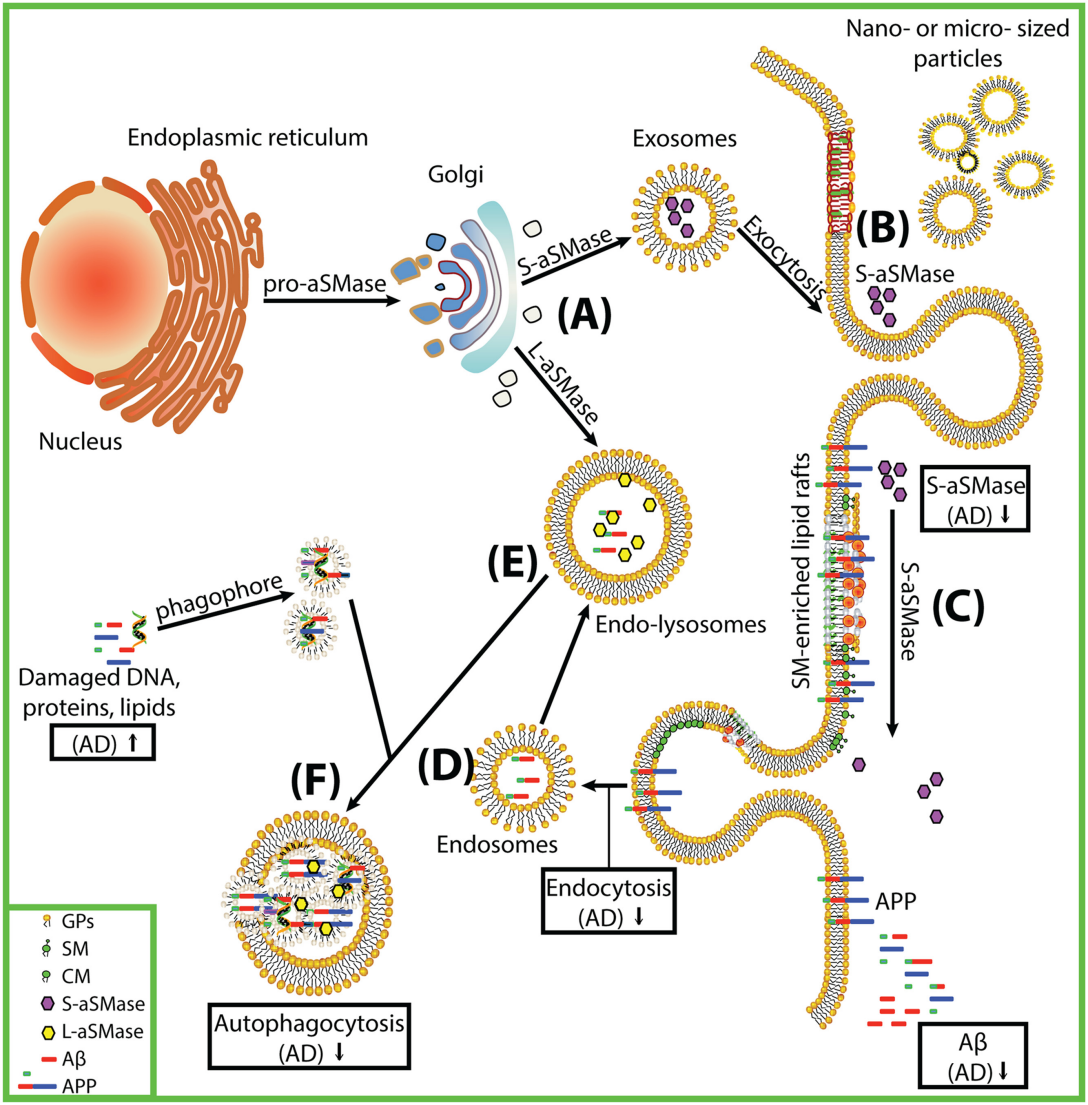
Scheme highlighting the role of perturbed sphingolipid metabolism in Alzheimer’s disease pathology. A) Pro-aSMase is distributed between the Golgi secretory pathway and the lysosomal pathway. B) SMase, ceramidase and phospholipases can induce the formation of secretory vesicles or blebbing of plasma membranes to generate nano- or micro-sized particles with different SP composition. C) Secreted aSMase (S-aSMase) plays a role in endocytosis, membrane repair or remodeling. D) Endocytosis mediated by aSMase influences amyloid precursor protein processing and the concentration of Aβ. E) Lysosomal aSMase (L-aSMase) plays a role in the fusion of phagophores with lysosomes, resulting in autophagocytosis (F). Any dysfunction in this sphingolipid metabolic pathway, such as the decrease in secretory aSMase in AD, will result in changes in the physical properties of cellular membranes, anomaly in exocytosis/endocytosis, defective trafficking of lipids and membrane-anchored proteins, resulting in dysfunctional membrane repair, and altered Aβ excretion. Abbreviations used on Fig 7. GP, glycerophospholipids; SM, sphingomyelin; Cer, ceramide; S_aSMase, secretory acid sphingomyelinase; L_aSMase, lysosomal acid sphingomyelinase; mb_nSMase, membrane bound neutral sphingomyelinase; APP, amyloid precursor protein.

Our data show that higher aSMase activity corresponds to higher Aβ_42_ in CSF from CN participants. Similarly, lower aSMase activity in the CSF from AD corresponds to lower Aβ_42_ levels compared with CN. aSMase activity significantly correlates with Aβ_42_ levels in CN but not in MCI or AD while no similar correlation is found for nSMase or to total tau protein. These data suggest that aSMase activity in CSF may be involved in the secretion of Aβ_42_ in health, thus preventing the accumulation of neurotoxic peptides in brain tissues. A scheme depicting how lower activities of aSMase may result in lower Aβ_42_ levels in CSF and higher plaque load in AD brain tissues is shown on Fig 7. This scheme suggests that dysfunction in SP metabolism in AD alters exocytosis/endocytosis at many sites. Amyloid precursor protein processing may be adversely affected in at least 6 locations: 7A) Pro-aSMase is distributed between the Golgi secretory pathway and the lysosomal pathway. Defective proteolytic maturation of SMase can lead to dysfunction and abnormal SM metabolism found in lipid storage disease [16]. This might result in the difference in sphingolipid metabolism that we report in CSF fractions from our AD subjects. The lower amounts of soluble aSMase found in AD will lower enzyme activity and compromise SM metabolism. 7B) Since aSMase [68,69], ceramidase [70] and phospholipase A_2_ [71–73] are implicated in secretory vesicle formation and membrane restructuring, decreased aSMase that we measured in AD may impact membrane remodeling and the generation of nano- or micro-sized particles, such as those in the NP fraction of CSF. Increased phospholipase A_2_ activity that we recently reported in CSF from AD subjects [41] may also modify membrane lipids. Any such modification of membrane composition will result in a shift in the physical properties of cellular membranes, anomaly in exocytosis, defective trafficking of lipids, and altered distribution of membrane-anchored proteins [74]. 7C) Secreted aSMase plays a role in endocytosis, membrane repair or remodeling [66]. Thus a decrease in aSMase that we measured in AD will reduce these processes and alter membrane properties and function. 7D) Brain aSMase and S1P levels correlate with amyloid beta peptide and hyperphosphorylated tau protein levels [75]. Both aSMase and nSMase mediated-endocytosis influence APP processing and thus regulate Aβ_42_ concentration [57,75], and influence membrane properties when they interact with components of lipid rafts. Lower aSMase activity in CSF from AD subjects will decrease APP processing and lower the ability of brain cells to clear toxic Aβ_42_. 7E) Lysosomal aSMase is important in autophagocytosis when phagophores fuse with lysosomes [76]. Less aSMase or improperly processed aSMase in AD will result in lowered endocytosis. 7F) aSMase plays a role in autophagocytosis, a process where denatured or oxidized DNA, lipids, and proteins such as Aβ_42_ that would otherwise be toxic to brain cells are digested and neutralized for resorption [77]. The lower aSMase activity in AD will result in less clearance of toxic products such as Aβ_42_, resulting in Aβ_42_ accumulation and neuronal death. In summary, a hallmark of AD pathology is the dysfunction of SP metabolism which impacts membrane remodeling and results in the abnormal clearance of neurotoxic Aβ_42_ peptides. Means of enhancing the secretion of Aβ_42_ from brain tissue may limit its accumulation and subsequent formation of neurotoxic plaques.

Dysfunction in SP metabolism in blood brain barrier cells may also alter their function. Disruption of pericyte structure compromises blood-brain-barrier function, and lowers amyloid clearance in an animal AD model [78]. Since SMase is involved in cell membrane repair, lowered aSMase activity that we report in AD would limit the ability of post-mitotic brain cells to repair after insults. In contrast, higher levels of aSMase in the CN population provide tools for better repair of damaged cell membranes. Approaches that prevent dysfunction in SP metabolism should result in better repair of post-mitotic neurons and regulation of crosstalk between apoptosis and autophagocytosis [79].

### Acid sphingomyelinase as a biomarker of cognitive function and potential for therapy

CSF Aβ_42_ is a reliable biomarker of AD and when combined with tau, the specificity for detecting AD is increased [80]. However, there is a need for other markers to be used with Aβ_42_ to increase diagnostic sensitivity and better understand AD pathophysiology. Our studies show that aSMase activity in CSF is sensitive in differentiating CN from AD and has a >90% value for predicting which subjects are CN. Thus, lower aSMase activity in CSF can also be utilized as a biomarker of AD.

### Exploring treatment implications from these results

Studies suggest that SMase is a multipurpose enzyme important in exocytosis, Aβ_42_ clearance, plasma membrane repair, and the control of SM levels [15,57,66]. Moreover, macrophages deficient in aSMase do not effectively traffic or efflux cholesterol while neurons lacking aSMase show anomaly in the distribution of glycosyl-anchored proteins [74,81]. Animal studies using recombinant aSMase show promise in regulating SM levels in lysosomal storage diseases [82]. Our results showing lower levels of aSMase in CSF from AD participants suggest that a similar approach to replace aSMase in AD may enhance Aβ_42_ secretion and clearance from the brain while stabilizing or repairing damaged neuronal plasma membranes.

### Conclusions

We show how sphingolipid metabolism differs between CSF fluid and particulate fractions and find a potential role of acid sphingomyelinase as an AD biomarker and a candidate therapeutic target. However, longitudinal studies are required to further evaluate whether the sphingolipid changes in CSF fractions are a cause or consequence of AD, whether they distinguish preclinical AD, or if they predict conversion to a more cognitively-impaired stage. More research into sphingolipid metabolism using different chromatographic approaches and higher resolution/accurate mass spectrometers is needed to identify specific ceramide and sphingomyelin species involved in AD pathology. These studies may reveal how compounds that attenuate ceramide toxicity, stabilize sphingolipid metabolism, and influence amyloid beta peptide clearance may be beneficial in AD prevention.

## Supporting Information

S1 Fig LC-MS/MS showing resolution of PC from SM. Top figure (A) shows the total ion current (TIC), MS/MS of PC(11:0/11:0) internal standard, precursor ion for 184 corresponding to synthetic PC(16:0/18:1) standard, and spectrum of PC(16:0/18:1). Bottom figure (B) shows TIC, MS/MS of PC(11:0/11:0) internal standard, precursor ion scan corresponding to bovine brain SM, and spectrum of SM.
(EPS)

S2 Fig Linearity of SP standard curves. Different amounts (0–50 ng) SP standards were mixed with 5 ng PC(11:0/11:0) internal standard. After LC-MS, peak ratios (area of SP standard divided by area of PC(11:0/11:0)) were calculated. Standard curves for Cer (S2A Fig), dhCer (S2B Fig), GlcCer (S2C Fig), and SM (S2D Fig) are representative of three separate experiments performed in duplicates.
(EPS)

S3 Fig Extracted ion chromatographs of internal standards using SRM (A) or Parent Ion Scan (B). (EPS)

S4 Fig Extracted ion chromatographs of SM species in CSF fractions. Representative SM species not influenced by isobars were extracted from LC-MS/MS chromatograph of SF and NP. S4 Fig is the total ion current (TIC), selected reaction monitoring (SRM) of PC(11:0/11:0) internal standard, and extracted ion chromatography of SM species in SF (S4A Fig) and NP (S4B Fig) fractions. These data are representative of fractions from 70 and 67 SF and NP participants, respectively.
(EPS)

S5 Fig Chromatographs and spectra of Cer and dhCer species in CSF fractions. Cer and dhCer species from LC-MS/MS were processed using the spectral feature of the Qual Browser software. S5 Fig shows representative spectra of Cer species in SF (S5A Fig, n = 70) and NP (S5B Fig, n = 67), and dhCer species in SF (S5C Fig, n = 70) and NP (S5D Fig, n = 67). For each Figure, the top and bottom spectra are derived from Peaks 1 and 2 from their respective chromatograph.
(EPS)

S1 Method Isolation of isobaric sphingomyelin species. (DOCX)

S2 Method Analyses of SP content in CSF fractions. (DOCX)

S3 Method Recovery. (DOCX)

S1 Table Sphingomyelin species in CSF. (DOC)

S2 Table SM species identified in SF fractions. (DOC)

S3 Table SM species identified in NP fraction. (DOC)

S4 Table Cer species identified in SF fraction. (DOC)

S5 Table Cer species identified in NP fraction. (DOC)

S6 Table dhCer species identified in SF fraction. (DOC)

S7 Table dhCer species identified in NP fraction. (DOC)
