# Supplementary material for: Sphingolipid Metabolism Correlates with Cerebrospinal Fluid Beta Amyloid Levels in Alzheimer’s Disease
Source: PLoS One. 2015 May 4;10(5):e0125597. doi: 10.1371/journal.pone.0125597 (PMC4418746; doi:10.1371/journal.pone.0125597)
Supplement: S1 Method — (DOCX) [file pone.0125597.s006.docx]

**S1 Method. Isolation of isobaric sphingomyelin species**. Isobaric interferences are a concern when identifying SM species found in CSF over a wide dynamic range and heterogeneity, so we reviewed spectral segments across the SM peak and then isolating only m/z that were not burdened by isobaric interference. Using this conservative approach, we showed that abundance of the isobars in our method was predictable with little variation and allowed confident identification of several SM species in CSF fractions (S1 Table).
