# Supplementary material for: Sphingolipid Metabolism Correlates with Cerebrospinal Fluid Beta Amyloid Levels in Alzheimer’s Disease
Source: PLoS One. 2015 May 4;10(5):e0125597. doi: 10.1371/journal.pone.0125597 (PMC4418746; doi:10.1371/journal.pone.0125597)
Supplement: S2 Method — (DOCX) [file pone.0125597.s007.docx]

**S2 Method. Analyses of SP content in CSF fractions.** Ideally, we would like to use a standard for each SP variant in CSF since these have been shown to yield slightly different signal responses for different species. While LIPID MAPS SP internal standards from Avanti Polar Lipids have been used for quantification, the discovery design (precursor ion scan) of our studies using HILIC (rather than MRM using reverse phase chromatography) precludes the use of similar standards because we do not know the SP species and their abundance in CSF fractions from normal and AD subjects, and our mass spectrometer may not differentiate co-migrating standards from unknown CSF SPs. While precursor scans using centroid mode was used for all SP classes, we used SRM in the profile mode for the IS since this gives > 6 fold higher signal response, with a single peak that is more symmetrical than the multiple peaks obtained in CSF samples using precursor ion scans (S2 Fig.). Moreover, this approach provided linear coefficients > 0.95 that suggests that it is can provide a reliable estimate of SP quantities in CSF fractions.
