# Supplementary material for: Sphingolipid Metabolism Correlates with Cerebrospinal Fluid Beta Amyloid Levels in Alzheimer’s Disease
Source: PLoS One. 2015 May 4;10(5):e0125597. doi: 10.1371/journal.pone.0125597 (PMC4418746; doi:10.1371/journal.pone.0125597)
Supplement: S3 Method — (DOCX) [file pone.0125597.s008.docx]

**S3 Method. Recovery**. SP standards and IS were suspended in 1 mL phosphate-buffered artificial CSF (<http://www.alzet.com/products/guide_to_use/cfs_> preparation.html) containing 0.25 mg/ml fatty acid free BSA. After lipid extraction and LC-MS as described, the amount of SPs was calculated to determine recovery.
