# Supplementary material for: Sphingolipid Metabolism Correlates with Cerebrospinal Fluid Beta Amyloid Levels in Alzheimer’s Disease
Source: PLoS One. 2015 May 4;10(5):e0125597. doi: 10.1371/journal.pone.0125597 (PMC4418746; doi:10.1371/journal.pone.0125597)
Supplement: S1 Table — (DOC) [file pone.0125597.s009.doc]

**S1 Table**. Sphingomyelin species in CSF

| **m/z** | **Intensity** | **m/z** | **Intensity** | **m/z** | **Intensity** | **m/z** | **Intensity** |
| --- | --- | --- | --- | --- | --- | --- | --- |
| 630.82 | 77680.24 | 720.58 | 10537.55 | 769.80 | 10847.76* | 813.86 | 1047124.97* |
| 631.05 | 2257.85 | 721.06 | 1627.61 | 770.65 | 10767.96 | 814.84 | 562048.69 |
| 634.49 | 3308.00 | 723.54 | 6797.12* | 771.69 | 15767.43* | 815.82 | 255534.50 |
| 635.66 | 1068.22 | 728.67 | 1609.88 | 772.93 | 15334.65 | 816.99 | 207915.56 |
| 636.25 | 9687.99 | 729.64 | 235210.49* | 773.77 | 50476.46* | 817.82 | 60179.87 |
| 647.65 | 1958.97 | 730.68 | 177147.57 | 774.81 | 62247.43 | 818.70 | 26536.41 |
| 648.58 | 413.39 | 731.72 | 1899193.04* | 775.74 | 26074.52 | 819.92 | 17559.20 |
| 650.53 | 1624.87 | 732.77 | 575146.65 | 776.50 | 4262.67 | 820.68 | 32668.25 |
| 673.52 | 928.16 | 733.85 | 245076.24 | 777.66 | 3276.51 | 821.93 | 19117.38 |
| 675.70 | 50449.91* | 734.91 | 92994.53 | 780.03 | 1108.30 | 822.90 | 14206.72 |
| 676.78 | 23042.18 | 735.76 | 77295.07 | 781.54 | 3014.69 | 823.79 | 21001.63* |
| 677.53 | 1407.05 | 736.89 | 38734.36 | 782.69 | 36023.23 | 824.73 | 602.21 |
| 678.65 | 1169.60 | 737.67 | 40086.52* | 783.87 | 17930.37 | 825.79 | 38305.22* |
| 679.86 | 6205.24* | 738.96 | 19467.42 | 784.73 | 17911.39 | 826.86 | 669.25 |
| 681.84 | 1117.56 | 739.39 | 10932.15 | 785.90 | 336770.82* | 827.85 | 36669.71* |
| 686.44 | 246.21 | 740.48 | 4610.19 | 787.00 | 119855.63 | 828.78 | 5102.23 |
| 689.69 | 69452.69* | 741.21 | 8658.39* | 787.87 | 325179.87* | 829.82 | 22134.99* |
| 692.68 | 28889.16 | 742.60 | 4527.31 | 788.73 | 138567.35 | 830.48 | 12829.54 |
| 693.33 | 8007.90 | 743.65 | 14136.74* | 789.94 | 61524.71 | 831.83 | 20669.87* |
| 695.94 | 308.35 | 744.53 | 9790.31 | 790.88 | 25149.79 | 832.83 | 41644.00 |
| 699.59 | 913.25 | 745.74 | 39837.31* | 791.98 | 39987.99* | 833.82 | 22768.38* |
| 700.43 | 991.17 | 746.86 | 20470.56 | 792.57 | 5170.52 | 834.84 | 17605.67 |
| 701.68 | 126605.75* | 747.81 | 7293.40 | 793.97 | 6766.55* | 835.71 | 3933.25 |
| 702.71 | 108396.54 | 748.60 | 119.05 | 795.80 | 5328.70 | 838.81 | 5213.54 |
| 703.81 | 1950627.48* | 749.73 | 21212.79* | 797.97 | 23906.56* | 839.58 | 1048.05 |
| 704.84 | 831854.34 | 750.26 | 2854.02 | 798.64 | 4403.42 | 840.93 | 25466.04 |
| 705.78 | 211485.43 | 754.52 | 978.06 | 799.74 | 146177.11* | 841.79 | 3282.80 |
| 706.59 | 98455.58 | 755.84 | 1966.39 | 800.74 | 35638.07 | 842.81 | 830.29 |
| 707.85 | 61509.86 | 756.70 | 4454.02 | 801.89 | 80201.68* | 843.66 | 4484.72* |
| 708.69 | 24775.08 | 757.75 | 83113.88* | 802.72 | 52956.33 | 848.77 | 724.03 |
| 709.70 | 47643.00* | 758.58 | 14343.17 | 803.80 | 35120.96 | 849.64 | 1624.38* |
| 710.08 | 29997.09 | 759.78 | 260733.98* | 804.61 | 6792.04 | 853.97 | 3503.11* |
| 711.25 | 30485.16* | 760.83 | 149205.40 | 805.73 | 12582.38* | 855.75 | 4326.00* |
| 712.90 | 1458.50 | 761.83 | 64866.37 | 806.78 | 8570.55 | 872.82 | 2872.90 |
| 713.95 | 14885.68* | 762.83 | 36623.52 | 807.80 | 25037.48* | 876.85 | 231.66 |
| 714.26 | 1308.94 | 764.70 | 6019.88 | 809.79 | 97774.39* | 885.75 | 685.37 |
| 717.76 | 49628.51* | 765.93 | 1177.23 | 810.85 | 32859.87 | 887.85 | 2143.20* |
| 718.77 | 35314.70 | 766.82 | 1582.46 | 811.80 | 373388.67* | 892.62 | 6298.62 |
| 719.78 | 2029.11 | 768.88 | 7128.43 | 812.95 | 205698.00 | 895.39 | 315.64 |

* Distinct sphingomyelin species not encumbered by isobaric peaks are representative of SF fractions from 70 cognitively healthy participants. A similar distribution was detected in the NP fractions.
