# Supplementary material for: Sphingolipid Metabolism Correlates with Cerebrospinal Fluid Beta Amyloid Levels in Alzheimer’s Disease
Source: PLoS One. 2015 May 4;10(5):e0125597. doi: 10.1371/journal.pone.0125597 (PMC4418746; doi:10.1371/journal.pone.0125597)
Supplement: S2 Table — (DOC) [file pone.0125597.s010.doc]

**S2 Table.** SM species identified in SF fractions

| **Input*^a^*  Mass** | **Matched  Mass** | **Delta*^b^*** | **C*^c^*** | **D.B.*^c^*** | **Abbreviation*^c^*** | **Formula** |
| --- | --- | --- | --- | --- | --- | --- |
| 647.60 | 647.5123 | 0.0924 | 12 | 0 | SM(d18:1/12:0) | C_35_H_72_N_2_O_6_P |
| 703.57 | 703.5749 | 0.0012 | 16 | 0 | SM(d18:1/16:0) | C_39_H_80_N_2_O_6_P |
| 705.59 | 705.5905 | 0.0025 | 16 | 0 | SM(d18:0/16:0) | C_39_H_82_N_2_O_6_P |
| 729.59 | 729.5905 | 0.0019 | 18 | 1 | SM(d18:1/18:1) | C_41_H_82_N_2_O_6_P |
| 731.60 | 731.6062 | 0.0105 | 18 | 1 | SM(d18:1/18:0) | C_41_H_84_N_2_O_6_P |
| 733.61 | 733.6218 | 0.0151 | 18 | 0 | SM(d18:0/18:0) | C_41_H_86_N_2_O_6_P |
| 759.66 | 759.6375 | 0.0244 | 20 | 0 | SM(d18:1/20:0) | C_43_H_88_N_2_O_6_P |
| 761.62 | 761.6531 | 0.0365 | 20 | 0 | SM(d18:0/20:0) | C_43_H_90_N_2_O_6_P |
| 787.67 | 787.6688 | 0.005 | 22 | 0 | SM(d18:1/22:0) | C_45_H_92_N_2_O_6_P |
| 789.62 | 789.6844 | 0.0599 | 22 | 0 | SM(d18:0/22:0) | C_45_H_94_N_2_O_6_P |
| 807.67 | 807.6375 | 0.0318 | 24 | 4 | SM(d18:1/24:4) | C_47_H_88_N_2_O_6_P |
| 809.68 | 809.6531 | 0.029 | 24 | 4 | SM(d18:0/24:4) | C_47_H_90_N_2_O_6_P |
| 813.69 | 813.6844 | 0.0047 | 24 | 1 | SM(d18:1/24:1) | C_47_H_94_N_2_O_6_P |
| 815.68 | 815.7001 | 0.0192 | 24 | 0 | SM(d18:1/24:0) | C_47_H_96_N_2_O_6_P |
| 817.80 | 817.7157 | 0.0838 | 24 | 0 | SM(d18:0/24:0) | C_47_H_98_N_2_O_6_P |
| 843.66 | 843.7314 | 0.0688 | 26 | 0 | SM(d18:1/26:0) | C_49_H_100_N_2_O_6_P |

*^a^*Precursor ion scans of 184 at a retention time that corresponded to SM species with intensity responses > 1000 to obtain input masses.

*^b^*Theoritical matched masses from Lipid Maps Mass Spectrometry prediction tool for sphingolipids (<http://www.lipidmaps.org/tools/ms/sphingolipids_batch.html>).

*^c^*Input m/z tolerance or delta defined as the difference between input m/s and matched m/z was set at 0.1.

*^d^*Structures (carbon number (C), double bonds (DB), abbreviated structure and formula) for SM species are representative of 70 CSF extracts from cognitively healthy study participants.
