# Supplementary material for: Sphingolipid Metabolism Correlates with Cerebrospinal Fluid Beta Amyloid Levels in Alzheimer’s Disease
Source: PLoS One. 2015 May 4;10(5):e0125597. doi: 10.1371/journal.pone.0125597 (PMC4418746; doi:10.1371/journal.pone.0125597)
Supplement: S3 Table — (DOC) [file pone.0125597.s011.doc]

**S3 Table.** SM species identified in NP fraction

| **Input  Mass** | **Matched  Mass** | **Delta*^a^*** | **C*^b^*** | **D.B*^b^*** | **Abbreviation*^b^*** | **Formula** |
| --- | --- | --- | --- | --- | --- | --- |
| 703.59 | 703.5749 | 0.011 | 16 | 0 | SM(d18:1/16:0) | C_39_H_80_N_2_O_6_P |
| 705.59 | 705.5905 | 0.0002 | 16 | 0 | SM(d18:0/16:0) | C_39_H_82_N_2_O_6_P |
| 729.59 | 729.5905 | 0.0053 | 18 | 1 | SM(d18:1/18:1) | C_41_H_82_N_2_O_6_P |
| 731.60 | 731.6062 | 0.0029 | 18 | 0 | SM(d18:1/18:0) | C_41_H_84_N_2_O_6_P |
| 733.74 | 733.6218 | 0.1147 | 18 | 0 | SM(d18:0/18:0) | C_41_H_86_N_2_O_6_P |
| 759.66 | 759.6375 | 0.0258 | 20 | 0 | SM(d18:1/20:0) | C_43_H_88_N_2_O_6_P |
| 761.67 | 761.6531 | 0.0194 | 20 | 0 | SM(d18:0/20:0) | C_43_H_90_N_2_O_6_P |
| 787.68 | 787.6688 | 0.007 | 22 | 0 | SM(d18:1/22:0) | C_45_H_92_N_2_O_6_P |
| 789.65 | 789.6844 | 0.0294 | 22 | 0 | SM(d18:0/22:0) | C_45_H_94_N_2_O_6_P |
| 809.72 | 809.6531 | 0.0622 | 24 | 4 | SM(d18:0/24:4) | C_47_H_90_N_2_O_6_P |
| 813.61 | 813.6844 | 0.0742 | 24 | 1 | SM(d18:1/24:1) | C_47_H_94_N_2_O_6_P |
| 815.70 | 815.7001 | 0.0016 | 24 | 1 | SM(d18:1/24:0) | C_47_H_96_N_2_O_6_P |
| 817.64 | 817.7157 | 0.0804 | 24 | 0 | SM(d18:0/24:0) | C_47_H_98_N_2_O_6_P |
| 841.68 | 841.7157 | 0.0388 | 26 | 1 | SM(d18:1/26:1) | C_49_H_98_N_2_O_6_P |
| 843.92 | 843.7314 | 0.1853 | 26 | 0 | SM(d18:1/26:0) | C_49_H_100_N_2_O_6_P |

*^a^*Input m/z tolerance or delta defined as the difference between input m/s and matched m/z was set at 0.2.

*^b^*C, DB for SM species in the NP fraction are are representative of 70 CSF extracts.
