# Supplementary material for: Sphingolipid Metabolism Correlates with Cerebrospinal Fluid Beta Amyloid Levels in Alzheimer’s Disease
Source: PLoS One. 2015 May 4;10(5):e0125597. doi: 10.1371/journal.pone.0125597 (PMC4418746; doi:10.1371/journal.pone.0125597)
Supplement: S4 Table — (DOC) [file pone.0125597.s012.doc]

**S4 Table.** Cer species identified in SF fraction

| **Input  Mass** | **Matched  Mass** | **Delta^a^** | **C** | **D.B.** | **Abbreviation** | **Formula** |
| --- | --- | --- | --- | --- | --- | --- |
| 564.78 | 564.535 | 0.2493 | 18 | 1 | Cer(d18:1/18:1(9Z)) | C_36_H_70_NO_3_ |
| 644.45 | 644.5096 | 0.057 | 12 | 0 | ^c^GlcCer(d18:1/12:0) | C_36_H_70_NO_8_ |
| 700.47 | 700.5722 | 0.101 | 16 | 0 | GlcCer(d18:1/16:0) | C_40_H_78_NO_8_ |
| 728.62 | 728.6035 | 0.0125 | 18 | 0 | GlcCer(d18:1/18:0) | C_42_H_82_NO_8_ |
| 762.89 | 762.7698 | 0.1191 | 32 | 0 | Cer(d18:1/32:0) | C_50_H_100_NO_3_ |
| 784.70 | 784.6661 | 0.0335 | 22 | 0 | GlcCer(d18:1/22:0) | C_46_H_90_NO_8_ |
| 804.82 | 804.6348 | 0.1866 | 24 | 4 | GlcCer(d18:1/24:4) | C_48_H_86_NO_8_ |
| 810.62 | 810.6818 | 0.0645 | 24 | 1 | GlcCer(d18:1/24:1(15Z)) | C_48_H_92_NO_8_ |
| 812.68 | 812.6974 | 0.0192 | 24 | 0 | GlcCer(d18:1/24:0) | C_48_H_94_NO_8_ |

*^a^* Input m/z tolerance or delta defined as the difference between input m/s and matched m/z was set at 0.25..

*^b^*C, DB for Cer species in the SF fraction are representative of 70 CSF extracts.

**^c^**Glucosylceramide and galactosylceramide isomers are not separated by our method.
