# Supplementary material for: Sphingolipid Metabolism Correlates with Cerebrospinal Fluid Beta Amyloid Levels in Alzheimer’s Disease
Source: PLoS One. 2015 May 4;10(5):e0125597. doi: 10.1371/journal.pone.0125597 (PMC4418746; doi:10.1371/journal.pone.0125597)
Supplement: S5 Table — (DOC) [file pone.0125597.s013.doc]

**S5 Table.** Cer species identified in NP fraction

| **Input  Mass** | **Matched  Mass** | **Delta*^a^*** | **C*^b^*** | **D.B.*^b^*** | **Abbreviation** | **Formula** |
| --- | --- | --- | --- | --- | --- | --- |
| 566.55 | 566.5507 | 0.0001 | 18 | 0 | Cer(d18:1/18:0) | C_36_H_72_NO_3_ |
| 650.63 | 650.6446 | 0.0149 | 24 | 0 | Cer(d18:1/24:0) | C_42_H_84_NO_3_ |
| 566.50 | 566.5507 | 0.0488 | 18 | 0 | Cer(d18:1/18:0) | C_36_H_72_NO_3_ |
| 648.54 | 648.6289 | 0.0875 | 24 | 1 | Cer(d18:1/24:1) | C_42_H_82_NO_3_ |
| 644.62 | 644.5096 | 0.1115 | 12 | 0 | GlcCer(d18:1/12:0) | C_36_H_70_NO_8_ |
| 734.97 | 734.7385 | 0.2282 | 30 | 0 | Cer(d18:1/30:0) | C_48_H_96_NO_3_ |
| 538.75 | 538.5194 | 0.232 | 16 | 0 | Cer(d18:1/16:0) | C_34_H_68_NO_3_ |
| 812.46 | 812.6974 | 0.2373 | 24 | 0 | ^c^GlcCer(d18:1/24:0) | C_48_H_94_NO_8_ |

*^a^*Input m/z tolerance or delta defined as the difference between input m/s and matched m/z was set at 0.25.

*^b^*C, DB for Cer species in the NP fraction are representative of 70 CSF extracts.

**^c^**Glucosylceramide and galactosylceramide isomers are not separated by our method.
