# Supplementary material for: Sphingolipid Metabolism Correlates with Cerebrospinal Fluid Beta Amyloid Levels in Alzheimer’s Disease
Source: PLoS One. 2015 May 4;10(5):e0125597. doi: 10.1371/journal.pone.0125597 (PMC4418746; doi:10.1371/journal.pone.0125597)
Supplement: S6 Table — (DOC) [file pone.0125597.s014.doc]

**S6Table.** dhCer species identified in SF fraction

| **Input  Mass** | **Matched  Mass** | **Delta*^a^*** | **C*^b^*** | **D.B.*^b^*** | **Abbreviation** | **Formula** |
| --- | --- | --- | --- | --- | --- | --- |
| 568.92 | 568.5663 | 0.3523 | 18 | 0 | Cer(d18:0/18:0) | C_36_H_74_NO_3_ |
| 644.31 | 644.5976 | 0.2887 | 24 | 4 | Cer(d18:0/24:4) | C_42_H_78_NO_3_ |
| 680.46 | 680.6915 | 0.2325 | 26 | 0 | Cer(d18:0/26:0) | C_44_H_90_NO_3_ |
| 736.87 | 736.7541 | 0.1202 | 30 | 0 | Cer(d18:0/30:0) | C_48_H_98_NO_3_ |
| 758.78 | 758.6505 | 0.1254 | 20 | 0 | ^c^GlcCer(d18:0/20:0) | C_44_H_88_NO_8_ |
| 793.21 | 792.8167 | 0.392 | 34 | 0 | Cer(d18:0/34:0) | C_52_H_106_NO_3_ |
| 812.43 | 812.6974 | 0.2638 | 24 | 1 | GlcCer(d18:0/24:1) | C_48_H_94_NO_8_ |

*^a^*Input m/z tolerance or delta defined as the difference between input m/s and matched m/z was set at 0.5.

*^b^*C, DB for Cer species in the SF fraction are representative of 70 CSF extracts.

**^c^**Glucosylceramide and galactosylceramide isomers are not separated by our method
