# Supplementary material for: Sphingolipid Metabolism Correlates with Cerebrospinal Fluid Beta Amyloid Levels in Alzheimer’s Disease
Source: PLoS One. 2015 May 4;10(5):e0125597. doi: 10.1371/journal.pone.0125597 (PMC4418746; doi:10.1371/journal.pone.0125597)
Supplement: S7 Table — (DOC) [file pone.0125597.s015.doc]

**S7 Table.** dhCer species identified in NP fraction

| **Input  Mass** | **Matched  Mass** | **Delta*^a^*** | **C*^b^*** | **D.B.*^b^*** | **Abbreviation** | **Formula** |
| --- | --- | --- | --- | --- | --- | --- |
| 540.57 | 540.535 | 0.0319 | 16 | 0 | Cer(d18:0/16:0) | C_34_H_70_NO_3_ |
| 566.59 | 566.5507 | 0.0398 | 18 | 1 | Cer(d18:0/18:1) | C_36_H_72_NO_3_ |
| 624.31 | 624.6289 | 0.3158 | 22 | 0 | Cer(d18:0/22:0) | C_40_H_82_NO_3_ |
| 644.72 | 644.5976 | 0.1185 | 24 | 4 | Cer(d18:0/24:4) | C_42_H_78_NO_3_ |
| 652.69 | 652.6602 | 0.027 | 24 | 0 | Cer(d18:0/24:0) | C_42_H_86_NO_3_ |
| 708.97 | 708.7228 | 0.2502 | 28 | 0 | Cer(d18:0/28:0) | C_46_H_94_NO_3_ |
| 736.53 | 736.7541 | 0.2258 | 30 | 0 | Cer(d18:0/30:0) | C_48_H_98_NO_3_ |
| 786.71 | 786.6818 | 0.0297 | 22 | 0 | ^c^GlcCer(d18:0/22:0) | C_46_H_92_NO_8_ |
| 812.24 | 812.6974 | 0.4611 | 24 | 1 | GlcCer(d18:0/24:1) | C_48_H_94_NO_8_ |
| 842.90 | 842.7444 | 0.1548 | 26 | 0 | GlcCer(d18:0/26:0) | C_50_H_100_NO_8_ |
| 926.36 | 926.8383 | 0.4772 | 32 | 0 | GlcCer(d18:0/32:0) | C_56_H_112_NO_8_ |

*^a^*Input m/z tolerance or delta defined as the difference between input m/s and matched m/z was set at 0.5.

*^b^*C, DB for Cer species in the NP fraction are representative of 70 CSF extracts.

**^c^**Glucosylceramide and galactosylceramide isomers are not separated by our method
